# Occupational risks associated with severe COVID-19 disease and SARS-CoV-2 infection – a Swedish national case-control study conducted from October 2020 to December 2021

**DOI:** 10.5271/sjweh.4103

**Published:** 2023-09-01

**Authors:** Kjell Torén, Maria Albin, Tomas Bergström, Nicola Murgia, Magnus Alderling, Linus Schiöler, Maria Åberg

**Affiliations:** 1 School of Public Health and Community Medicine, Institute of Medicine, Sahlgrenska Academy, University of Gothenburg, Gothenburg, Sweden.; 2 Occupational and Environmental Medicine, Department of Medicine, Sahlgrenska University Hospital, Gothenburg, Sweden.; 3 Division of Occupational and Environmental Medicine, Lund University, Lund, Sweden.; 4 Unit of Occupational Medicine, Institute of Environmental Medicine, Karolinska Institutet, Stockholm, Sweden.; 5 Department of Infectious Diseases/Virology, Institute of Biomedicine, University of Gothenburg, Gothenburg, Sweden.; 6 Department of Environmental and Prevention Sciences, University of Ferrara, Ferrara, Italy.; 7 Region Västra Götaland, Regionhälsan, Gothenburg, Sweden.

**Keywords:** coronavirus, epidemiology, JEM, job exposure matrix, occupation, workplace

## Abstract

**Objective:**

This study aimed to investigate whether workplace factors and occupations are associated with SARS-CoV-2 infection or severe COVID-19 in the later waves of the pandemic.

**Methods:**

We studied 552 562 cases with a positive test for SARS-CoV-2 in the Swedish registry of communicable diseases, and 5985 cases with severe COVID-19 based on hospital admissions from October 2020 to December 2021. Four population controls were assigned the index dates of their corresponding cases. We linked job histories to job-exposure matrices to assess the odds for different transmission dimensions and different occupations. We used adjusted conditional logistic analyses to estimate odds ratios (OR) for severe COVID-19 and SARS-CoV-2 with 95% confidence intervals (CI).

**Results:**

The highest OR for severe COVID-19 were for: regular contact with infected patients, (OR 1.37, 95% CI 1.23–1.54), close physical proximity (OR 1.47, 95% CI 1.34–1.61), and high exposure to diseases or infections (OR 1.72, 95% CI 1.52–1.96). Mostly working outside had lower OR (OR 0.77, 95% CI 0.57–1.06). The odds for SARS-CoV-2 when mostly working outside were similar (OR 0.83, 95% CI 0.80–0.86). The occupation with the highest OR for severe COVID-19 (compared with low-exposure occupations) was certified specialist physician (OR 2.05, 95% CI 1.31–3.21) among women and bus and tram drivers (OR 2.04, 95% CI 1.49–2.79) among men.

**Conclusions:**

Contact with infected patients, close proximity and crowded workplaces increase the risks for severe COVID-19 and SARS-CoV-2 infection. Outdoor work is associated with decreased odds for SARS-CoV-2 infection and severe COVID-19.

Occupational exposures and dimensions are important determinants of respiratory infections (1). This has been evident in the coronavirus disease (COVID-19) pandemic caused by infection with severe acute respiratory syndrome coronavirus 2 (SARS-CoV-2) (2). Several studies have identified higher risk levels for different occupational groups, mainly healthcare and transportation workers, as well as occupations that involve personal service duties (3–7). These studies have indicated that workers who come in close contact with either the general public or infected patients are at increased risk for COVID-19. However, few studies have analyzed the importance of different occupation-related contact modalities. In a British study, it has been shown that workers who were in close contact daily with others had a higher prevalence of antibodies against SARS-CoV-2, as compared to homeworkers (8). Several job-exposure matrices (JEM) have been developed to assess workplace factors that are associated with exposure to SARS-CoV-2 (9–11).

The majority of the studies conducted to date have focused on the first wave of the pandemic, and only a few have examined the development of occupational risk in the subsequent waves of the pandemic. There are probably considerable differences between the waves depending on the extent of implementation of preventive measures, which include improved access to appropriate personal protection equipment, adherence to disease-prevention guidelines, and expanded vaccination programs. In addition, more-contagious SARS-CoV-2 variants emerged, increasing viral transmission in society (12, 13). The extent of this may also differ between occupational groups (14).

It is of importance to emphasize that the course of the pandemic and its associated occupational risks should be discussed in the specific context of a given country. Sweden differs from many other countries in that there were less-strict rules regarding social distancing and the use of face masks, and schools were not closed (15). During the first wave of the pandemic, there was selected screening of SARS-CoV-2 for healthcare workers, which is why analyses of occupational risks based on positive tests for SARS-CoV-2 might be biased for that period.

We hypothesized that, even in the later waves of the pandemic, workers who were in close contact with either the general public or with infected patients had increased odds for severe COVID-19 and SARS-CoV-2 infection.

## Methods

### Establishment of the study population

The study population included as cases all persons with a positive polymerase chain reaction (PCR) test for the SARS-CoV-2 virus, with the information being obtained through the system of mandatory reporting of communicable diseases in Sweden, the SmiNet registry, as previously reported (16). We restricted the cases that were eligible for inclusion to subjects aged 18–64 years and to reports that were received between 1 October 2020 and 31 December 2021 (N=683 566). This was a period of widespread testing for the virus in society, without any focus on certain occupations in the second and third waves of the pandemic. From the SmiNet registry, we extracted the Swedish personal identity number of each case and the date (index date) when the positive PCR sample was obtained. We selected four living controls for each case, matched for gender, age (case year of birth) and region of residency on the index date, from the Swedish Historical National Population Registry (N=3 404 166).

We extracted information from the Swedish national socioeconomic database, called LISA (the longitudinal integration database for health insurance and labor market studies), regarding the highest educational level attained [categorized as: pre-high school (up to 9 years), completed high school, or university examination]; country of birth; and dwelling-area including the number of inhabitants in the residence. From LISA, we also obtained information about annual occupational history for the period of 2014–2020. Finally, we included only those cases (N=561 582) and corresponding controls (N=2 211 372) for whom there was information regarding occupation during the period of 2014–2020.

Among the cases, we defined two separate types: for SARS-CoV-2, all persons with a positive PCR test for SARS-CoV-2 (N=561 582) and, for severe COVID-19, the cases with a positive PCR test for SARS-CoV-2 and admission to hospital for a COVID-19-related diagnosis sometime in the period from 7 days prior to the index date to 30 days after the index date. We also included deceased persons who had diagnosis of COVID-19 (U07.1 or U07.2) as an underlying cause-of-death up to 90 days after the index date. This resulted in a final population of 5985 cases with severe COVID-19. A COVID-19-related diagnosis was defined as a definitive diagnosis where three infectious disease specialists had listed it as a COVID-19-related disease *and* a diagnosis of U07.1 or U07.2 (17). These diagnoses were obtained from the Swedish National Hospital Discharge Registry and the Swedish National Mortality Register (supplementary material, www.sjweh.fi/article/4103, table S1).

### Comorbidities

We used the Swedish National Hospital Discharge Registry and the Swedish Prescribed Drug Registry to identify the following comorbidities as confounders based on their ICD-10 codes: chronic obstructive pulmonary disease (COPD, ICD10 J43-J44); ischemic heart disease (IHD, ICD10 I20-I25); and diabetes mellitus (ICD E10-E14) during the three years preceding the index date. We defined the use of oral and systemic corticosteroids according to the Anatomical Therapeutic Chemical (ATC) codes (ATC H02) if these drugs were dispensed at any time within one year preceding the index date.

### Classification of occupational exposures

The occupation of each individual in the year closest preceding the index date, in 2014–2020, was classified at the four-digit level according to the ISCO-88 and ISCO-08 codes (18, 19). We applied two previously described JEM to assess the risk of becoming infected with the SARS-CoV-2 virus: an international (9) and Swedish (10) JEM, as previously described (20). The international JEM was designed to capture eight dimensions that were judged to be important for the risk of being infected, divided into the categories of low, increased, and high risk. All the dimensions were compared with the 'no risk' category – defined as homeworkers or persons not working with others. We used the Danish application of the JEM, as it was assumed to reflect Swedish conditions. Thus, in the present study, we applied the five following categories of risk dimensions: (i) number of workers in close vicinity to each other per day: high (>30), increased (10–30), and low (<10) risk; (ii) nature of contacts: high [working in workspaces with regular contacts with persons with suspected or diagnosed COVID-19 (for this application, infected patients)], increased (working with the general public), and low (working in workspaces with coworkers only) risk; (iii) contaminated workspaces: high (frequently sharing materials/surfaces with the general public ≥10 times/day), medium (sometimes sharing materials/surfaces with the general public <10 times/day), and low (frequently sharing materials/surfaces with coworkers ≥10 times /day) risk; (iv) working location: high (mostly inside for >4 hours/day), medium (partly inside for 1–4 hours/day), and low (mostly outside) risk; (v) social distancing, ie, the possibility to maintain ≥1 m of social distance: high (can never be maintained), increased (cannot always be maintained), and low (can always be maintained) risk.

We also applied a Swedish JEM, based on the O*NET data, which maps physical proximity and exposure to diseases or infections, as previously described (10, 20). This JEM has standardized scores for each occupational group, yielding scores in the range of 0–100.

Thus, the scale for physical proximity was:

0 – I do not work near other people (>30 m distance);
25 – I work with others but not in close proximity (eg, private office);
50 – I work in slightly close proximity (eg, shared office) to other persons;
75 – I work in moderately close proximity (at arm’s length) to other persons; and
100 – I work in very close proximity (near touching) to other persons.

The scale for Daily exposure to diseases or infections at the current workplace was as follows:

0–24 – At least once a year, but not every month; 1^st^ group.
25–49 – At least once a month, but not every week; 2^nd^ group
50–74 – At least once a week, but not daily; 3^rd^ group
75–100 – Daily; 4^th^ group, highest exposure.

We present the results in four groups of the mean scores for each dimension.

### Statistical methods

We used a conditional logistic multivariable regression analysis to calculate the odds for a positive test for SARS-CoV-2 and for severe COVID-19 in association with the JEM-defined categories of exposures tested as indicator variables. The basic (matched) models were adjusted only for matching strata (ie, equivalent to adjusting for gender, age and geographic region, and index date). For SARS-CoV-2 infection, we present only the matched model. The further-adjusted models included, in addition, education, country of birth, location/inhabitants of the residence, number of inhabitants of the residence, chronic obstructive pulmonary disease (COPD), ischemic heart disease (IHD), diabetes, and dispensed corticosteroids. These confounders were selected a priori but underlying assumptions are visualized in a DAG model (supplementary figure S1). All the JEM-defined exposure categories were tested in separate models for each exposure. We also analyzed the interactions by stratification with regard to gender and metropolitan area (Stockholm).

Furthermore, we analyzed the risks for SARS-CoV-2 infection and severe COVID-19 disease for all the occupations (4-digit level), using >500 cases (SARS-CoV-2 infection) and >50 cases (severe COVID-19). The reference group in this analysis was comprised of occupations that were classified as having the lowest level of potential exposure using the international and Swedish JEM (ie, the three most common occupations were commercial sales representatives, software developers, and advertising and marketing professionals). Regarding SARS-CoV-2 infection, the occupations were tested in unconditional matched models with adjustments for gender, age and geographic region. For severe COVID-19 disease the occupations were tested in the adjusted models (see above). We also performed gender stratified analyses for the different occupations. In the stratified analysis, we used regarding severe COVID-19 >25 cases and for SARS-CoV-2 infection >250 cases. SARS-CoV-2 infection we only report the 20 occupations with the highest and lowest odds ratios (OR), respectively.

All the statistical analyses were performed using the SAS version 9.4 M7 software (SAS Inc, Cary, NC, USA), and 95% confidence intervals (CI) were calculated.

## Results

The study comprised 561 582 cases of SARS-CoV-2 infection and 5985 cases with severe COVID-19 disease. Of the cases with severe COVID-19, 121 were deceased within 90 days of the index date. Descriptive data for the cases and corresponding controls, including the prevalences of occupational exposures, are described in table 1.

**Table 1:** Characteristics of cases with severe COVID-19 disease and SARS-CoV-2 infection and matched controls from the general population of Sweden in the age range of 18–64 years. [COPD=chronic obstructive pulmonary disease.]

	Severe COVID-19 disease N=5985		Controls N=24 315		SARS-CoV-2 infection N=561 582		Controls N=2 211 372
	% (N)		% (N)		% (N)		% (N)
Men	57.3 (3432)		57.7 (14 028)		48.8 (274 317)		49.1 (1 086 046)
Born in Sweden	61.5 (3678)		80.7 (19 615)		78.3 (439 713)		80.1 (1 771 830)
Post-high school	39.5 (2367)		43.8 (10 639)		43.8 (246 223)		45.3 (1 002 192)
COPD ^a^	0.8 (48)		0.2 (40)		0.1 (510)		0.1 (2367)
Diabetes mellitus^a^	6.0 (362)		2.1 (508)		1.5 (8 278)		1.5 (33 066)
Ischemic heart disease^a^	2.7 (164)		1.0 (237)		0.5 (8 278)		0.5 (11 026)
Dispensed corticosteroids	10.8 (645)		3.9 (943)		3.5 (19 491)		3.3 (72 139)
Workers (>30) in close proximity to each other	28.4 (1 702)		22.1 (5 375)		29.3 (164 741)		24.6 (543 416)
Regular contacts with infected patients	17.1 (1024)		12.3 (2 995)		16.4 (91 826)		13.8 (305,090)
Frequently sharing materials / surfaces with the general public	43.3 (2592)		33.2 (8 073)		42.4 (238 047)		37.3 (825 858)
Working mostly inside	73.8 (4416)		66.1 (16 062)		72.0 (404 224)		68.1 (1 505 305)
Social distancing can never be maintained	26.4 (1579)		19.4 (4705)		25.5 (143 464)		22.1 (487 850)
Work very close to other persons	27.7 (1658)		19.5 (4739)		26.2 (146 995)		22.0 (485 560)
Daily exposure to diseases or infections	7.4 (443)		5.0 (1202)		6.6 (37 113)		5.3 (116 461)

^a^ Hospital-based diagnoses.

For severe COVID-19 disease, the highest odds in the matched models were seen for the dimensions of: regular contact with infected patients (OR 1.86, 95% CI 1.68–2.05), physical proximity (OR 1.86, 95% CI 1.71–2.03), and the highest exposure group of exposure to diseases or infections (OR 1.87, 95% CI 1.66–2.10) (table 2). In the additionally adjusted models, the odds were generally lower but the pattern was similar. The highest odds were still for regular contact with infected patients (OR 1.37, 95% CI 1.23–1.52, physical proximity (OR 1.47, 95% CI 1.34–1.61), and the highest exposure group of exposure to diseases or infections (OR 1.72, 95% CI 1.52–1.96). The odds were moderately increased for frequently sharing materials/surfaces with general public (OR 1.30, 95% CI 1.19–1.41) and the odds were decreased (although the confidence interval comprised unity) for mostly working outside (OR 0.77, 95% CI 0.57–1.06).

**Table 2:** Conditional logistic multivariable regression models for severe COVID-19 disease and SARS-CoV-2 infection in relation to the different dimensions of transmission and mitigation factors. [OR=odds ratio; CI=confidence interval.]

Transmission and mitigation factors		Severe COVID-19 disease N=5985				SARS-CoV-2 infection N=561 582
		Matched ^a^		Adjusted ^b^		Matched ^a^
		OR (95% CI)		OR (95% CI)		OR (95% CI)
Number of workers in close proximity to each other (per day)						
	<10	1.10 (1.01–1.19)		0.95 (0.87–1.03)		1.01 (1.00–1.02)
	10–30	1.54 (1.41–1.68)		1.20 (1.09–1.32)		1.21 (1.19–1.22)
	>30	1.69 (1.55–1.84)		1.36 (1.24–1.49)		1.36 (1.35–1.37)
Nature of contacts						
	In workspaces with coworkers only	1.13 (1.04–1.22)		0.98 (0.90–1.07)		1.07 (1.06–1.08)
	In workspaces with the general public	1.51 (1.39–1.64)		1.23 (1.13–1.35)		1.22 (1.21–1.23)
	Regular contacts with infected patients	1.86 (1.68–2.05)		1.37 (1.23–1.52)		1.37 (1.35–1.38)
Contaminated workspaces						
	Frequently sharing materials/surfaces with coworkers (≥10 times/day)	1.12 (1.03–1.21)		1.00 (0.92–1.09)		1.09 (1.08–1.10)
	Sometimes sharing materials/surfaces with the general public (<10 times/day)	1.10 (0.94–1.28)		1.04 (0.88–1.22)		1.01 (1.00–1.03)
	Frequently sharing materials/surfaces with the general public (≥10 times/day)	1.71 (1.58–1.85)		1.30 (1.19–1.41)		1.28 (1.27–1.29)
Work location						
	Mostly outside	0.90 (0.67 – 1.21)		0.77 (0.57 – 1.06)		0.83 (0.80 – 0.86)
	Partly inside	0.94 (0.82–1.08)		0.84 (0.72–0.97)		1.04 (1.02–1.05)
	Mostly inside	1.43 (1.33–1.53)		1.17 (1.08–1.26)		1.19 (1.18–1.20)
Social distancing						
	Always maintained	1.17 (1.08–1.27)		1.05 (0.96–1.14)		1.09 (1.08–1.09)
	Not always possible	1.33 (1.22–1.45)		1.09 (0.99–1.20)		1.18 (1.17–1.19)
	Never maintained	1.77 (1.62–1.93)		1.32 (1.21–1.45)		1.29 (1.28–1.31)
Physical proximity						
	3^rd^ versus 2^nd^ and 1^st^	1.21 (1.12–1.30)		1.12 (1.04–1.21)		1.13 (1.12–1.14)
	4^th^ versus 2^nd^ and 1^st^	1.86 (1.71–2.03)		1.47 (1.34–1.61)		1.38 (1.37–1.40)
Exposure to diseases or infections						
	2^nd^ versus 1^st^	1.48 (1.38–1.60)		1.22 (1.13–1.32)		1.13 (1.12–1.14)
	3^rd^ versus 1^st^	1.68 (1.66–2.10)		1.44 (1.31–1.59)		1.42 (1.41–1.44)
	4^th^ versus 1^st^	1.87 (1.66–2.10)		1.72 (1.52–1.96)		1.44 (1.42–1.45)

^a^ Models matched for age, gender, and region. ^b^ Models matched for age, gender, region, and adjusted for education, country of birth, dwelling area/inhabitants, number of inhabitants in the dwelling, chronic obstructive pulmonary disease, ischemic heart disease, diabetes, and dispensed corticosteroids.

For SARS-CoV-2 infection, in the matched models, the highest odds were for the dimensions of regular contact with infected patients (OR 1.37, 95% CI 1.35–1.38), physical proximity (OR 1.38, 95% CI 1.37–1.40), and for the highest exposure group of exposure to diseases or infections (OR 1.44, 95% CI 1.42–1.45) (Table 2). The odds for SARS-CoV-2 infection were decreased for mostly working outside (OR 0.83, 95% CI 0.80–0.86).

The results were similar for men and women, both regarding severe COVID-19 disease (table 3) and SARS-CoV-2 infection (supplementary table S2). We also separately analyzed the metropolitan area of Stockholm (the capital city) and found similar results (data not shown).

**Table 3:** Conditional logistic multivariable regression models of severe COVID-19 disease among men and women in relation to the different dimensions of transmission and mitigation factors. [OR=odds ratio; CI=confidence interval.]

Dimensions of transmission and mitigation factors		Severe COVID-19 N=5985		
		Men N=3376		Women N=2523
		OR (95% CI) ^a^		OR (95% CI) ^a^
Number of workers in close proximity to each other (per day)				
	<10	0.91 (0.82–1.01)		1.03 (0.89–1.21)
	10–30	1.13 (1.00–1.29)		1.31 (1.13–1.52)
	>30	1.28 (1.13–1.46)		1.47 (1.29–1.67)
Nature of contacts				
	In workspaces with coworkers only	0.93 (0.93–1.03)		1.10 (0.94–1.28)
	In workspaces with the general public	1.21 (1.07–1.36)		1.30 (1.13–1.48)
	Regular contacts with infected patients	1.23 (1.02–1.47)		1.50 (1.30–1.72)
Contaminated workspaces				
	Frequently sharing materials/surfaces with coworkers (≥10 times/day)	0.92 (0.83–1.02)		1.22 (1.05–1.41)
	Sometimes sharing materials/surfaces with the general public (<10 times/day)	1.06 (0.85–1.34)		1.04 (0.81–1.32)
	Frequently sharing materials/surfaces with the general public (≥10 times/day)	1.26 (1.12–1.41)		1.37 (1.21–1.56)
Work location				
	Mostly outside	0.75 (0.54–1.04)		0.71 (0.29 – 1.74)
	Partly inside	0.84 (0.71–0.98)		0.58 (0.36 – 0.94)
	Mostly inside	1.07 (0.97–1.19)		1.32 (1.17–1.49)
Social distancing				
	Always maintained	0.97 (0.87–1.09)		1.19 (1.04–1.37)
	Not always possible	0.99 (0.88–1.12)		1.30 (1.11–1.52)
	Never maintained	1.29 (1.12–1.47)		1.42 (1.24–1.62)
Physical proximity				
	3^rd^ versus 2^nd^ and 1^st^	1.03 (0.94–1.14)		1.31 (1.14–1.50)
	4^th^ versus 2^nd^ and 1^st^	1.31 (1.15–1.50)		1.72 (1.49–1.97)
Exposure to diseases or infections				
	2^nd^ versus 1^st^	1.22 (1.09–1.36)		1.23 (1.08–1.39)
	3^rd^ versus 1^st^	1.27 (1.05–1.52)		1.52 (1.35–1.72)
	4^th^ versus 1^st^	1.83 (1.45–2.30)		1.71 (1.47–2.01)

^a^ Models matched for age and region.

Table 4 lists the odds for severe COVID-19 disease in all the occupations with >50 cases, as compared with the unexposed control occupations. The five occupations with the highest odds for severe COVID-19 disease were bus and tram drivers, nursing professionals, primary school teachers, early childhood educators and childcare workers. Of note is that heavy truck and lorry drivers did not have increased odds for severe COVID-19. Table 5 lists the odds for the 20 occupations with the highest odds for SARS-CoV-2 infection. The five occupations with the highest odds for SARS-CoV-2 infection were prison guards, early childhood educators, primary school teachers, firefighters, and midwifes. The 20 occupations with the lowest OR for SARS-CoV-2 infection are listed in supplementary table S3.

**Table 4:** Odds ratios (OR) for severe COVID-19 disease in all occupations with ≥50 cases. OR are shown with 95% confidence intervals (CI) relative to occupations with low exposure.

Occupation	ISCO 2008 No	Cases (N)	OR ^a^	95% CI
Bus and tram drivers	8331	98	1.98	1.48–2.66
Nursing professionals	2221	122	1.68	1.33–2.12
Primary school teachers	2341	177	1.60	1.31–1.96
Early childhood educators	2342	110	1.72	1.35–2.19
Childcare workers	5311	165	1.60	1.31–1.96
Certified specialist physicians	2211	76	1.53	1.14–2.05
Health care assistants	5321	486	1.46	1.25–1.71
Teachers’ aides	5312	71	1.43	1.05–1.91
Elementary workers, not elsewhere classified	9629	97	1.43	1.07–1.91
Taxi drivers	8322	73	1.32	0.96–1.81
Home-based personal care workers	5322	228	1.21	0.99–1.47
Kitchen helpers	9412	117	1.18	0.91–1.54
Policy administration officials	2422	70	1.14	0.86–1.50
Cooks	5120	74	1.11	0.83–1.49
Metalworking tool setters	7223	81	1.09	0.82–1.43
Motor vehicle mechanics	7231	53	1.09	0.78–1.52
Shop sales assistants	5223	170	0.99	0.81–1.21
Domestic cleaners	9111	155	0.90	0.72–1.13
Heavy truck and lorry drivers	8332	90	0.94	0.72–1.22
Building caretakers	5153	86	0.97	0.74–1.27
Production clerks	4322	110	0.77	0.60–0.98

^a^ Models are matched for age, gender, and region, and further adjusted for education, country of birth, dwelling area/inhabitants, number of inhabitants in the dwelling, chronic obstructive pulmonary disease, ischemic heart disease, diabetes, and dispensed corticosteroids.

**Table 5:** Odds ratios (OR) for SARS-CoV-2 infection in occupations with >500 cases. OR are shown with 95% confidence intervals (CI) relative to occupations with low exposure among the 20 occupations with the highest OR values.

Occupation	ISCO 2008 No	Cases (N)	OR ^a^	95% CI
Prison guards	5413	987	1.70	1.60–1.81
Early childhood educators	2342	14 187	1.69	1.65–1.72
Primary school teachers	2341	18 319	1.67	1.64–1.70
Firefighters	5411	946	1.66	1.54–1.79
Midwifes	2222	837	1.60	1.48–1.73
Teachers’ aides	5312	7116	1.59	1.55–1.64
Childcare workers	5311	16 466	1.58	1.55–1.61
Childcare service managers	1341	648	1.58	1.45–1.73
Police officers	5412	2574	1.58	1.51–1.65
Nursing professionals	2221	13 774	1.56	1.53–1.60
Education methods specialists	2351	1868	1.54	1.46–1.62
Health services managers	1342	2362	1.54	1.51–1.56
Pharmaceutical technicians	3213	605	1.54	1.40–1.69
Healthcare assistants	5321	39 886	1.54	1.51–1.56
Social work professionals	3412	3800	1.51	1.45–1.57
Hair dressers	5141	2686	1.46	1.39–1.52
Restaurant managers	1412	1301	1.45	1.36–1.55
Certified specialist physicians	2211	5489	1.45	1.41–1.50
Education mangers (head teachers)	1345	1677	1.45	1.37–1.53
Dental assistants	3251	1956	1.44	1.36-1.51

^a^ Models are matched for gender, age, and region.

The odds for severe COVID-19 among men and women are shown in supplementary table S4. Among men the highest odds were for bus and tram drivers (OR 2.04, 95% CI 1.49–2.79), security guards and elementary workers. Among women the highest odds were for certified specialized physician (OR 2.05, 95% CI 1.31–3.21), early childhood educators, and nursing professionals. The odds for SARS-CoV-2 infection among men and women are shown in supplementary tables S5 and S6.

## Discussion

In the present study with national coverage, we show that during the second and third waves of the pandemic, close contact with infected or diseased patients/persons and close physical proximity still increased the odds of having severe COVID-19 disease, as well as having been infected with the SARS-CoV-2 virus. Outdoor work seems to be protective against infection. The observed pattern among the occupations supports the notion that contact with infected patients/persons and close proximity persist as important risk factors.

A major strength of our study design is that we used national databases with high levels of coverage to assess the outcomes of interest: SARS-CoV-2 infection and severe COVID-19 disease. We also utilized a more-specific definition of severe COVID-19 disease compared with most other studies. In addition to an in-hospital diagnosis and the COVID-19-related U07 diagnosis, we required a diagnosis that was clinically linked to COVID-19 disease (17). This will diminish the risk of having false positive associations as we avoid to classify as cases, comorbidity not clearly related to COVID-19, ie, persons with unrelated diseases and accidentally detected COVID-19. We used the SmiNet registry, which has comprehensive coverage of all SARS-CoV-2 tests in Sweden, although we acknowledge that not all cases with positive detection of the virus will be captured. We also use the Swedish Inpatient Register, which is acknowledged to be of high quality (21). However, we acknowledge that the diagnoses may be misclassified, but as we studied the younger part of the population (<65 years) we consider that this misclassification will not severely bias the results. Another strength of our study is that we employed random controls from the same national population. Furthermore, we were able to consider a number of key potential confounders using Swedish registry data. These confounders included level of education (as a proxy for socioeconomic status, SES), living density in dwellings, and comorbidities that might modify the risk, such as diabetes, COPD, IHD and dispensed corticosteroids. Despite these adjustments, we cannot exclude a residual bias from, for instance, smoking habits or use of public transportation.

We did not control for COVID-19 vaccination. However, in the latter part of our study period, in December 2021, 80.5% of the adult population had received two doses of COVID-19 vaccine. A study from the County of Stockholm has analyzed the age-standardized prevalence of vaccination against COVID-19 in different occupations covering the period until January 2022 (22). Among women, the high-risk occupations, certified specialized physician, early childhood educators and nursing professionals had rather high prevalence of vaccination: 86.0%, 75.7% and 82.9%, respectively. Among men, the prevalences in high-risk occupations were lower: bus and tram drivers (63.7%), security guards (76.5%) and elementary workers (66.6%). From these data, it is difficult to conclude whether the vaccinations were effective in protecting workers at risk. Another limitation regarding the occupational analyses is the false positive (or negative) results due to multiple testing. Hence, the results regarding the different occupations have to be regarded as hypothesis generating results.

A key analytic strength of this study is our approach to categorizing occupational exposure. It is generally acknowledged that the JEM approach avoids the recall bias inherent to respondent-elicited exposure histories (23). Furthermore, we limited the analyses to occupational exposures during the year preceding the diseased state as we assumed that this period was critical in terms of increased risk. The Swedish JEM for proximity and exposure to diseases is based on data collected in the US (24). We do not consider this to be a problem, besides which US military personnel were not included. Military personnel constitute a very small fraction of the Swedish working-age population. We do not include the dimension of face covering, which has been applied differently in Sweden compared to many other countries. In addition, we do not include the dimensions of income insecurity or migrant background. The reasons for this are twofold. First, we have data on socioeconomic status and migrant status on the individual level from our national registries. Second, we use the Danish application of the international JEM, and we consider that the Swedish and Danish labor markets are different with regards to migration and income security.

We performed the JEM analyses based on assumptions of exposure to infected persons and the risk of close proximity. The subsequent analyses of a high number of occupations may have resulted in some spurious associations of increased or decreased risk for some occupations due to random variation, regardless of the presence of statistical significance. However, we may have missed some occupations that are at risk, due to the low numbers, ie, occupations with <50 persons with COVID-19 disease or <500 SARS-CoV-2-positive persons. However, in the gender stratified analyses we used <25 persons and <250 persons as the limit. Therefore, cautious interpretation and critical discussions are needed before the results for specific occupations are communicated (3).

The main outcomes of the present study are that close contacts in the workplace (ie, physical proximity and contact with infected or diseased persons or patients, increase the risks for severe COVID-19 and a positive test for SARS-CoV-2. Even if this is in line with inferences drawn from studies that have looked at specific occupations, few studies have investigated these dimensions in such detail. In a British study, workers who had close daily contacts with others were more likely to be seropositive for SARS-CoV-2 compared to homeworkers (8). The results of meta-analyses also support the idea that physical distancing in general decreases the incidences of SARS-CoV-2 infection and COVID-19 (25). In a recent study that applied the British version of the international JEM, associations were noted between SARS-CoV-2 infection and the number of contacts and social distancing, and the authors observed that in three domains – number, nature of contacts, and social distancing – there was an exposure–response relationship between the exposure levels and risk of SARS-CoV-2 infection (26). In a British study that applied the O*NET-based JEM, frequent occupational exposure to disease/infections and working in close proximity with others were associated with increased risk for a positive SARS-CoV-2 test (27).

Taken together, our results and those of previous studies support the reasonable conclusion that close physical proximity in the workplace and contacts with infected/diseased persons/patients increase the risks for a positive test for SARS-CoV-2 and the clinical disease of COVID-19.

In the present study, frequently sharing materials or surfaces with the general public did not appear to be a strong risk factor. In the British application of the international JEM, the importance of sharing surfaces was also uncertain, showing either a lack of dose–response or no increase in risk (26). The SARS-CoV-2 virus is often detected on surfaces, although whether or not these viruses are viable remains uncertain (28). This may explain the unclear results with regard to this dimension. In our study, outdoor work seems to be protective. However, in other studies, the converse has been found. In the British application of the international JEM, outdoor work was associated with increased odds for SARS-CoV-2 infection (26). In a US study, outdoor workers had higher odds for SARS-CoV-2 infection compared to indoor workers (29).

One possible mechanism for the observed inconsistencies with regard to risk from outdoor work may be that although the virus concentration is generally likely to be attenuated in the larger air volumes, these workers may to a varying degree share high-risk indoor facilities eg, for breaks and change of clothes. Hence, our observation may be highly dependent on such specific circumstances and may not be applicable to other contexts.

Thus, our observations have to be replicated to remove uncertainty regarding the evidence.

Our findings regarding different occupations and risks for both SARS-CoV-2 infection and severe COVID-19 disease are broadly consistent with the reported results from smaller studies conducted in other countries and in other contexts. Our results showing increased risks for nurses, midwifes and certified specialist physicians corroborate earlier studies reporting increased risks among healthcare workers (3, 4, 6, 8, 30, 31). Other occupations that involve close contacts with both the general public and infected persons are taxi drivers and bus drivers. In a Chinese study, using a taxi more than once a week was a clear risk for severe acute respiratory syndrome (SARS) (32). Infected persons may use public transportation; a British study noted that during the influenza season of 2008–2009, patients attending their primary care physician more often had used the bus or tram prior to coming in contact with their physician, as compared with controls (33). This may represent a way for bus/tram drivers to be infected. We found an almost doubled odds for severe COVID-19 disease among bus and tram drivers, although such a relationship to a positive test for SARS-CoV-2 was not found among these workers. In an Italian study, (especially male) bus drivers had almost a three-fold increased risk of COVID-19 (34). A similar occupation, albeit with less contact with the public, is heavy truck and lorry drivers. This occupation did not show increased odds for severe COVID-19. Therefore, we conclude that bus and tram drivers are probably at increased risk for severe COVID-19 disease due to occupational exposure. We also observed increased odds, both for severe COVID-19 and SARS-CoV-2 infection, among primary school teachers and early childhood educators. In Sweden, both elementary schools and kindergartens remained open during the pandemic, and with some exceptions so did the upper secondary schools. The Swedish COVID-19 Commission concluded that it was a clear benefit for the children and for society that schools were kept open (15). We agree with this, although the increased risk of disease for the front-line workers in primary schools and kindergartens underscores the need for the introduction of additional safety measures to reduce viral transmission and to maintain a high frequency of vaccination in these occupational groups. Teachers in primary schools and kindergartens are mostly in the age interval groups for which the vaccination rates are rather low.

The gender stratification of occupations clearly showed that among women the occupations in health care and childcare were associated with increased risk: certified specialist physicians, early childhood educators, nursing professionals and childcare workers. Among men the increased risk was for other kind of social services, like bus and tram drivers, security guards, elementary workers and kitchen helpers.

We conclude that close contacts with infected or diseased patients/persons and close physical proximity increase the odds of having a positive test for SARS-CoV-2 virus or for suffering from severe COVID-19 disease. The findings for different occupations support the hypothesis that contacts with infected patients/persons and close proximity are important risk factors. The results indicate that occupational groups outside the healthcare sector also may be considered for occupational compensations. There is a need to introduce additional safety measures, including vaccinations, to reduce viral transmission in these work environments.

### Ethics approval

The Gothenburg Ethics Committee approved the study (Dnr- 04792-19).

## Supplementary material

Supplementary material

## Data Availability

The study outcomes are based on matching with Swedish national registers. Data are available upon reasonable request. Requesters for data sharing need to have a Swedish ethical permit.
