# Supplementary material for: Occupational risks associated with severe COVID-19 disease and SARS-CoV-2 infection – a Swedish national case-control study conducted from October 2020 to December 2021
Source: Scand J Work Environ Health. 2023 Aug 29;49(6):386–94. doi: 10.5271/sjweh.4103 (PMC10789521; doi:10.5271/sjweh.4103)
Supplement: Supplementary material [file SJWEH-49-386-S001.pdf]

Occupational risks associated with severe COVID-19 disease and SARS-CoV-2 infection – a Swedish national case-control study conducted from October 2020 to December 2021[1]

by Kjell Torén, MD,[2] Maria Albin, MD, Tomas Bergström, MD, Nicola Murgia, MD, Magnus Alderling, PhD, Linus Schiöler, PhD, Maria Åberg, MD

1. Supplementary Material
2. Correspondence to: Kjell Torén, School of Public Health and Community Medicine, The Sahlgrenska Academy, University of Gothenburg, Box 414, SE-405 30 Gothenburg, Sweden. [E-mail: Kjell.Toren@amm.gu.se].

Table S1. List of included principal diagnoses among cases with severe COVID-19 (U07.1 or U07.2) as a principal diagnosis in the National Patient Register.

Included categories	ICD-code
**Symptom**	
Cough	R05
Abnormalities of breathing	R06
Other symptoms or signs involving the circulatory and respiratory systems	R09
Dizziness and giddiness	R42
Unknown fever	R50
Headache	R51
Malaise and fatigue	R53
Syncope and collapse	R55
**Respiratory tract infections**	
Influenza	J10
Viral and bacterial pneumonias	J12-J13, J15-J18
Unspecified acute lower respiratory infection	J22
Coronavirus infection, unspecified	B34.2, B97.2
Other viral infections	B34.8 - B34.9
Other and unspecified infectious diseases	B99
**Respiratory disorders**	
Pulmonary embolism	I26
Acute respiratory distress syndrome	J80

Pulmonary edema	J81
Pleural effusion, not elsewhere classified	J90
Respiratory failure, not elsewhere classified	J96
Respiratory disorders in diseases classified elsewhere	J99
**Obstructive airway diseases**	
Acute bronchitis	J20
Acute bronchiolitis	J21
Chronic obstructive pulmonary disease	J44
Asthma	J45
**Cardiac diseases**	
Viral carditis	B33.2
Acute pericarditis	I30
Pericarditis in diseases classified elsewhere	I32
Chronic ischemic heart disease	I25
Myocarditis	I40-I41
Atrial fibrillation and flutter	I48
Heart failure	I50
Abnormalities of heart beat	R00
**Electrolyte disorders**	
Other disorders of fluid, electrolyte and acid-balance	E87

Table S2. Conditional logistic multivariable regression models of SARS-CoV-2 infection among men and women in relation to the different dimensions of transmission and mitigation factors. Models are matched for age, gender and region.

**Dimensions of transmission and mitigation factors**	**SARS-CoV-2 infection N= 561 582**	
	**Men N=273 957**	**Women N=287 265**
	**OR (95% CI)**	**OR (95% CI)**
**Number of workers in close proximity to each other**		
<10 per day	1.01 (1.00–1.02)	1.00 (0.98–1.01)
10–30 per day	1.13 (1.12–1.15)	1.27 (1.26–1.29)
>30 per day	1.30 (1.28–1.32)	1.40 (1.39–1.42)
**Nature of contacts**		
In workspaces with coworkers only	1.04 (1.03–1.05)	1.09 (1.08–1.11)
In workspaces with the general public	1.16 (1.14–1.17)	1.27 (1.25–1.28)
Regular contacts with infected patients	1.35 (1.32–1.37)	1.40 (1.38–1.41)
**Contaminated workspaces**		
Frequently sharing materials/surfaces with coworkers (≥10 times/day)	1.04 (1.03–1.05)	1.16 (1.14–1.17)
Sometimes sharing materials/surfaces with the general public (<10 times/day)	1.03 (1.00–1.06)	1.01 (0.99–1.03)
Frequently sharing materials/surfaces with the general public (≥10 times/day)	1.23 (1.21–1.24)	1.33 (1.31–1.34)
**Location**		
Mostly working outside	0.82 (0.79–0.85)	0.75 (0.70–0.81)
Working partly inside	1.02 (1.00–1.03)	0.93 (0.90–0.96)
Working mostly inside	1.12 (1.11–1.13)	1.27 (1.26–1.28)
**Social distancing**		
Always maintained	1.02 (1.01–1.04)	1.15 (1.14–1.17)
Not always	1.10 (1.09–1.12)	1.28 (1.26–1.30)
Never maintained	1.27 (1.25–1.29)	1.34 (1.33–1.36)
**Physical proximity**		
3rd vs. 2nd and 1st	1.10 (1.09–1.11)	1.18 (1.17–1.19)
4th vs. 2nd and 1st	1.34 (1.32–1.36)	1.44 (1.42–1.45)

**Exposure to diseases or infections**		
2nd vs. 1st	1.08 (1.07–1.10)	1.17 (1.15–1.18)
3rd vs. 1st	1.47 (1.45–1.50)	1.42 (1.40–1.43)
4th vs. 1st	1.48 (1.44–1.52)	1.43 (1.41–1.45)

Table S3. Odds ratios (ORs) for SARS-CoV-2 infection in occupations with >500 cases. ORs are shown with 95% confidence intervals (CI) relative to occupations with low exposure among the 20 occupations with the lowest OR values.

Occupation	ISCO 2008 No	Cases (N)	OR[a]	95% CI
Livestock and dairy producers	6121	955	0.62	0.58-0.67
University teachers	2310	2 479	0.62	0.58-0.67
Mixed crop and animal producers	6130	631	0.66	0.61-0.72
Librarians	2622	609	0.73	0.67-0.79
Gardeners	6113	1 735	0.76	0.73-0.80
Electronics engineer	2152	2 741	0.81	0.77-0.84
Chemical science technicians	3111	865	0.81	0.75-0.87
Heavy truck and lorry drivers	8332	5 199	0.82	0.79-0.84
Earth moving and related plant operators	8342	1 936	0.86	0.82-0.91
Wood processing plant operators	8172	704	0.87	0.81-0.95
Mechanical engineers	2144	1 705	0.89	0.84-0.93
Pulp and paper mill process workers	8171	809	0.90	0.84-0.97
Building construction laborer	9313	832	0.90	0.84-0.98
Garbage and recycling collectors	9611	941	0.93	0.86-0.99
Electrical engineers technicians	3113	605	0.93	0.89-0.97
Policy administration professionals	2422	5 877	0.94	0.91-0.97
Domestic cleaners	9111	9 310	0.94	0.92-0.96
Armed forces (civilian occupations)	0310	668	0.95	0.87-1.03
Engineering professionals NEC	2149	2 474	0.95	0.91-0.99
Civil engineers	2142	1 489	0.95	0.90-1.00

[a]Models are matched for gender, age, and region.

Table S4. Odds ratios (ORs) for severe COVID-19 disease in all occupations with ≥25 cases in either men or women. ORs are shown with 95% confidence intervals (CI) relative to occupations with low exposure.

Occupation	ISCO 2008 No	Men			Women		
		Cases (N)	OR[a]	95% CI	Cases (N)	OR[a]	95% CI
Bus and tram drivers	8331	92	2.04	1.49-2.79	N.a.	N.a.	N.a.
Security guards	5414	28	1.84	1.14-2.96	N.a.	N.a.	N.a.
Nursing professionals	2221	N.a.	N.a.	N.a.	104	1.81	1.37–2.39
Elementary workers, not elsewhere classified	9629	66	1.57	1.09-2.24	31	1.36	0.83–2.24
Kitchen helpers	9412	68	1.56	1.08-2.24	49	0.99	0.67–1.45
Primary school teachers	2341	62	1.51	1.09-2.11	115	1.74	1.33–2.28
Early childhood educators	2342	N.a.	N.a.	N.a.	95	1.84	1.38–2.45
Childcare workers	5311	N.a.	N.a.	N.a.	148	1.67	1.28–2.18
Food machine operators	8160	25	1.49	0.89-2.50	N.a.	N.a.	N.a.
Mining supervisors	3121	34	1.44	0.95-2.18	N.a.	N.a.	N.a.
Shopkeepers	5221	29	1.32	0.82-2.13	N.a.	N.a.	N.a.
Teachers' aides	5312	27	1.32	0.79-2.19	44	1.67	1.11–2.51
Taxi drivers	8322	66	1.23	0.87-1.72	N.a.	N.a.	N.a.
Home-based personal care workers	5322	75	1.22	0.89-1.67	153	1.26	0.96–1.64
Certified specialist physicians	2211	42	1.21	0.82-1.79	34	2.05	1.31–3.21
Health care assistants	5321	86	1.21	0.91-1.62	399	1.65	1.33–2.05
Metalworking tool setters	7223	71	1.10	0.81-1.48	N.a.	N.a.	0.82–1.43
Motor vehicle mechanics	7231	51	1.09	0.77-1.54	N.a.	N.a.	0.78–1.52
Building caretakers	5153	80	1.07	0.72-1.57	N.a.	N.a.	0.74–1.27
Cooks	5120	44	1.07	0.72-1.57	30	1.23	0.79–1.94
Agricultural machinery mechanics	7233	40	1.07	0.73-1.56	N.a.	N.a.	N.a.

Policy administration officials	2422	30	1.03	0.69-1.55	40	1.29	0.88–1.89
Heavy truck and lorry drivers	8332	87	0.98	0.75-1.29	N.a.	N.a.	0.72–1.22
Plumbers	7126	47	0.96	0.68-1.35	N.a.	N.a.	N.a.
Shop sales assistants	5223	78	0.96	0.72-1.27	92	1.08	0.81–1.44
Domestic cleaners	9111	53	0.96	0.67-1.38	101	0.90	0.66–1.22
Painters	7131	25	0.95	0.59-1.52	N.a.	N.a.	N.a.
Production clerks	4322	98	0.89	0.68-1.16	N.a.	N.a.	0.60–0.98
Social work and councelling professionals	2635	N.a.	N.a.	N.a.	30	1.08	0.69-1.67
Data entry clerks	4132	N.a.	N.a.	N.a.	62	1.31	0.94-1.82
Engineering professionals not elsewhere classified	2149	N.a.	N.a.	N.a.	25	0.99	0.63-1.56
Electrical engineering technicians	3113	32	0.85	0.57-1.28	N.a.	N.a.	N.a.
Electricians	7411	29	0.85	0.56-1.29	N.a.	N.a.	N.a.
Assemblers not elsewhere classified	8219	25	0.76	0.48-1.21	N.a.	N.a.	N.a.
House builders	7111	47	0.69	0.49-0.97	N.a.	N.a.	N.a.

[a]Models are matched for age, gender, and region, and further adjusted for education, country of birth, dwelling area/inhabitants, number of inhabitants in the dwelling, chronic obstructive pulmonary disease, ischemic heart disease, diabetes, and dispensed corticosteroids.

Table S5. Odds ratios (ORs) for SARS-CoV-2 infection among men in occupations with >250 cases. ORs are shown with 95% confidence intervals (CI) relative to occupations with low exposure among the 20 occupations with the highest OR values and the 20 occupations with the lowest OR values.

Occupation	ISCO 2008 No	Cases (N)	OR[a]	95% CI
**The twenty occupations with the highest OR values**				
Atlethes and sports player	3421	407	3.14	2.75-3.58
Prison guards	5413	952	1.83	1.70–1.98
Social work professionals	3412	1 543	1.81	1.70–1.92
Driving instructors	5165	306	1.75	1.53-2.00
Primary school teachers	2341	4 924	1.73	1.67–1.79
Early childhood educators	2342	1 449	1.72	1.61–1.83
Firefighters	5411	892	1.69	1.56–1.83
Nursing professionals	2221	1 709	1.65	1.56–1.75
Health services managers	1342	563	1.64	1.49–1.80
Police officers	5412	1 701	1.62	1.53–1.71
Physiotherapeuts	2264	438	1.61	1.45-1.80
Sports coaches, instructors and officials	3422	873	1.58	1.46-1.71
Metal production process controllers	3135	281	1.57	1.37-1.80
Teachers' aides	5312	2 784	1.56	1.49–1.64
Certified specialist physician	2211	2 638	1.53	1.46-1.60
Restaurant managers	1412	809	1.50	1.38-1.63
Financial and insurance services branch managers	1346	420	1.49	1.34-1.67
Education managers (head teachers)	1345	541	1.49	1.35-1.64
Healthcare assistants	5321	6 352	1.49	1.44–1.53
Hair dressers	5141	440	1.47	1.32-1.64
**The twenty occupations with the lowest OR values**				
Forestry workers	6210	323	0.56	0.50-0.63
Livestock and dairy producers	6121	571	0.58	0.53-0.63
University teachers	2310	1 258	0.61	0.58-0.65
Messengers, delivereres and luggage porters	9621	368	0.61	0.55-0.69
Field crop and vegetable growers	6111	336	0.62	0.55-0.70
Mobile farm and forestry workers	8341	470	0.63	0.57-0.69
Mixed crop and animal producers	6130	495	0.68	0.62-0.75
Gardeners	6113	1 226	0.75	0.71-0.80
Chemical science technicians	3111	415	0.79	0.71-0.88
Mixed crop and livestock farm labourers	9213	264	0.80	0.70-0.91
Heavy truck and lorry drivers	8332	4 821	0.81	0.79-0.84
	2152	2 315	0.82	0.78-0.85

Earth moving and related plant operators	8342	1 864	0.85	0.81-0.90
Wood processing plant operators	8172	624	0.85	0.78-0.93
Domestic cleaners	9111	2 330	0.88	0.84-0.92
Packing and labelling machine operators	8183	312	0.88	0.78-0.99
Building construction laborer	9313	789	0.89	0.83-0.97
Mechanical engineers	2144	1 291	0.90	0.84-0.95
Elementary workers, not elsewhere classified	9629	1 700	0.90	0.85-0.95
Pulp and paper mill process workers	8171	687	0.90	0.83-0.98
Garbage and recycling collectors	9611	829	0.92	0.85-0.99
Electrical engineers technicians	3113	2 539	0.94	0.90-0.99
Contact center informations clerks	4222	1 544	0.94	0.91-0.97
Armed forces (civilian occupations)	0310	600	0.94	0.86-1.03
Locomotive engine drivers	8311	386	0.95	0.85-1.06
Electrical line installers and repairers	7413	311	0.95	0.84-1.08

[a]Models are matched for age and region.

Table S6. Odds ratios (ORs) for SARS-CoV-2 infection among women in occupations with >250 cases. ORs are shown with 95% confidence intervals (CI) relative to occupations with low exposure among the 20 occupations with the highest OR values and with the lowest OR values.

Occupation	ISCO 2008 No	Cases (N)	OR[a]	95% CI
**The twenty occupations with the highest OR values**				
Early childhood educators	2342	12 738	1.69	1.65–1.73
Primary school teachers	2341	13 395	1.65	1.61–1.69
Teacher's aides	5312	4 332	1.61	1.55-1.67
Child care services managers	1341	611	1.61	1.47-1.77
Child care workers	5311	14 857	1.61	1.57-1.65
Midwifes	2222	836	1.60	1.47-1.73
Pharmaceutical technicians	3213	542	1.55	1.41-1.71
Nursing professionals	2221	12 065	1.55	1.51–1.59
Healthcare assistants	5321	33 534	1.55	1.52–1.58
Education methods specialists	2351	1 640	1.54	1.46-1.63
Service managers not elsewhere classified	1439	363	1.53	1.36-1.72
Prison guards	5413	952	1.83	1.70–1.98
Social work professionals	3412	1 543	1.81	1.70–1.92
Health services managers	1342	563	1.64	1.49–1.80
Hair dressers	5141	440	1.47	1.32-1.64
Dental assistants	3251	1 900	1.43	1.36-1.51
Education managers	1345	1 136	1.43	1.34-1.53
Agricultural machinery mechanics	7233	298	1.43	1.26-1.63
Dentists	2261	539	1.42	1.29-1.57
Restaurant managers	1412	492	1.38	1.25-1.53
**Occupations with the lowest OR values**				
University teachers	2310	1 221	0.67	0.63-0.72
Livestock and dairy producers	6121	384	0.70	0.63-0.78
Electronic enegineers	2152	2 315	0.77	0.69-0.85
Librarians	2622	489	0.77	0.70-0.85
Gardeners	6113	509	0.78	0.71-0.86
Heavy truck and lorry drivers	8332	378	0.79	0.71-0.88
Tax officials	3352	294	0.82	0.72-0.92
Electrical engineers technicians	3113	359	0.82	0.74-0.92
Chemical science technicians	3111	450	0.83	0.75-0.91
Mechanical engineering technicians	3115	552	0.84	0.77-0.92
Mechanical engineers	2144	414	0.86	0.77-0.95

Physical and engineering technicians not elsewhere classified	3119	559	0.87	0.80-0.96
Engineering professionals NEC	2149	734	0.91	0.84-0.99
Civil engineers	2142	412	0.91	0.82-1.01
Policy administration professionals	2422	3 727	0.92	0.89-0.95
Architects	2161	404	0.94	0.84-1.04
Contact centre sales persons	5244	408	0.95	0.85-1.05
Governments regulatory professionals not elsewhere classified	3359	1 270	0.95	0.90-1.01

[a]Models are matched for age, and region.

Fig S1. Visualization of DAG model
